# The carcinogenic effect of various multi-walled carbon nanotubes (MWCNTs) after intraperitoneal injection in rats

**DOI:** 10.1186/s12989-014-0059-z

**Published:** 2014-11-20

**Authors:** Susanne Rittinghausen, Anja Hackbarth, Otto Creutzenberg, Heinrich Ernst, Uwe Heinrich, Albrecht Leonhardt, Dirk Schaudien

**Affiliations:** Fraunhofer Institute for Toxicology and Experimental Medicine ITEM, Nikolai-Fuchs-Str. 1, 30625 Hannover, Germany; Leibniz Institute for Solid State and Materials Research Dresden, PF 270116, 01171 Dresden, Germany

**Keywords:** Carbon nanotubes, Carcinogenicity, Intraperitoneal injection, Mesothelioma, Nanotoxicology, Rats, MWCNT

## Abstract

**Background:**

Biological effects of tailor-made multi-walled carbon nanotubes (MWCNTs) without functionalization were investigated *in vivo* in a two-year carcinogenicity study. In the past, intraperitoneal carcinogenicity studies in rats using biopersistent granular dusts had always been negative, whereas a number of such studies with different asbestos fibers had shown tumor induction. The aim of this study was to identify possible carcinogenic effects of MWCNTs. We compared induced tumors with asbestos-induced mesotheliomas and evaluated their relevance for humans by immunohistochemical methods.

**Methods:**

A total of 500 male Wistar rats (50 per group) were treated once by intraperitoneal injection with 10^9^ or 5 × 10^9^ WHO carbon nanotubes of one of four different MWCNTs suspended in artificial lung medium, which was also used as negative control. Amosite asbestos (10^8^ WHO fibers) served as positive control. Morbid rats were sacrificed and necropsy comprising all organs was performed. Histopathological classification of tumors and, additionally, immunohistochemistry were conducted for podoplanin, pan-cytokeratin, and vimentin to compare induced tumors with malignant mesotheliomas occurring in humans.

**Results:**

Treatments induced tumors in all dose groups, but incidences and times to tumor differed between groups. Most tumors were histologically and immunohistochemically classified as malignant mesotheliomas, revealing a predominantly superficial spread on the serosal surface of the abdominal cavity. Furthermore, most tumors showed invasion of peritoneal organs, especially the diaphragm. All tested MWCNT types caused mesotheliomas. We observed highest frequencies and earliest appearances after treatment with the rather straight MWCNT types A and B. In the MWCNT C groups, first appearances of morbid mesothelioma-bearing rats were only slightly later. Later during the two-year study, we found mesotheliomas also in rats treated with MWCNT D – the most curved type of nanotubes. Malignant mesotheliomas induced by intraperitoneal injection of different MWCNTs and of asbestos were histopathologically and immunohistochemically similar, also compared with mesotheliomas in man, suggesting similar pathogenesis.

**Conclusion:**

We showed a carcinogenic effect for all tested MWCNTs. Besides aspect ratio, curvature seems to be an important parameter influencing the carcinogenicity of MWCNTs.

## Background

Discussions about the hazardous potential of CNTs began very early, with regard to possible toxic and carcinogenic effects well known from persistent natural and man-made mineral fibers. CNTs came under suspicion, because the generally accepted prerequisites for a mineral fiber to be able to exert a carcinogenic effect in the lung and pleura can also be found in some CNTs. These prerequisites include: low solubility, long alveolar macrophage clearance half-time, a fiber length of at least 5 μm and a fiber diameter of less than 3 μm with an aspect ratio (ratio of fiber length to fiber diameter) of at least 3:1. Fibers fulfilling these criteria fall under the definition of the so-called “WHO fibers” [1]. Experimental animal studies have also shown that the toxic and carcinogenic potency of mineral fibers increases with growing fiber length [2]. Fibers longer than 15 to 20 μm exerted stronger toxic and carcinogenic effects than shorter ones, because alveolar macrophages are unable to completely take up such longer fibers [3]. Epidemiological studies and also investigations in experimental animals have proven the toxic and carcinogenic effects of natural mineral fibers such as asbestos [4,5]. Fiber-specific tumors are mesotheliomas of the pleura, but lung tumors are caused as well. Man-made mineral fibers (MMMF) displaying low solubility, high biopersistence, and fulfilling the WHO fiber definition caused lung tumors and mesotheliomas in experimental animals in a way very similar to asbestos fibers [6].

Proving or disproving the carcinogenic potential of mineral fibers in experimental animals after inhalation exposure is very costly and there are only few laboratories worldwide that are able to adequately conduct such inhalation studies with fiber dusts. Therefore, another test was developed and accepted by the regulatory authorities in the EU to investigate whether newly developed mineral fibers have a potential to induce tumors originating from the epithelial cells of the pleura or peritoneum [7]. For this test, a suspension of a certain number concentration of WHO fibers of the mineral fiber under evaluation is injected once into the peritoneum of rats, and the animals thus treated have to be kept for two years and investigated histopathologically for mesotheliomas. The number of WHO fibers that have to be applied according to the regulatory authorities is 10^9^ and in a more rigorous test approach can be increased to 5 × 10^9^. Asbestos fibers that caused mesotheliomas in humans as well as asbestos fibers and man-made mineral fibers that caused mesotheliomas in experimental animals after inhalation exposure also caused mesotheliomas after intraperitoneal (i.p.) injection in rats [8]. This test in rats is specific for biopersistent fibers; biopersistent granular dusts, in contrast, are not able to induce mesotheliomas in this test system, even when administered at very high doses (4 × 20 mg per rat i.p.) [9].

CNTs can be produced as single- or multi-walled tubes. Diameters of single-walled carbon nanotubes (SWCNTs) are generally below 10 nm, whereas those of MWCNTs are mostly above 20 nm up to 150 nm [10,11]. In the present study, we investigated only MWCNTs. SWCNTs and also MWCNTs can be very long, reaching dimensions comparable to long mineral fibers, and CNTs are almost insoluble because of their inorganic carbon structure. This means that some MWCNTs also meet the prerequisites for causing fiber-specific toxic effects.

Early pathological effects observed after short-time intraperitoneal administration of CNTs in mice were very similar to effects observed with asbestos fibers [12]. Takagi and co-workers [13] found mesotheliomas in p53-heterozygous mice after intraperitoneal injection of CNTs and asbestos fibers, whereas a different study revealed no tumors in rats after i.p. injection of CNTs shorter than 1 μm [14]. In another study in rats, mesotheliomas were found after i.p. injection of MWCNTs with an average length of 4 to 5 μm [15].

In the present study, we also used the i.p. carcinogenicity test in rats to investigate whether four specially designed MWCNTs of different lengths, diameters, and curvatures have the potential to induce mesotheliomas similarly to asbestos fibers. We tested each of the four CNTs used with WHO dimensions at two CNT number concentrations according to the recommendation for the carcinogenicity test with mineral fibers. Fifty male rats were treated in each dose group. The negative control group was treated with suspension medium only and the positive control group with amosite asbestos at a ten-fold lower fiber number. We chose the number concentration used for i.p. administration of amosite asbestos based on published data showing that 1 × 10^8^ WHO amosite asbestos fibers will induce about 50% mesothelioma-bearing rats after two years of experimental time [16].

Besides the number and percentage of MWCNTs with WHO dimensions, we also determined the percentage of MWCNTs longer than 20 μm per injected suspension.

For suspension of the nanotubes and also for the negative control group, we used the fluid according to Porter and co-workers [17] so as to prevent aggregation of nanotubes. Due to the content of 1,2-dipalmitoyl-sn-glycero-3-phosphocholine this fluid has similarities to surfactant.

Experimental animals were killed when morbid or after two years of experimental time. We performed histopathological examination and determined the numbers of animals with mesotheliomas per experimental group. In addition, we conducted immunohistochemical investigations of the tumor tissue in respect of some markers specific for mesothelioma. Furthermore, we also recorded body weight and mortality.

The tumors induced by intrapleural or intraperitoneal injection of asbestos fibers in rats were classified as malignant mesotheliomas [18,19] closely resembling human malignant mesothelioma in their biological behavior and histopathology. Human malignant mesotheliomas have been described to show epithelial, sarcomatoid, or biphasic growth patterns [20,21]. The same categories have been specified for rat mesotheliomas [22]. For mesotheliomas in humans, a variety of immunohistochemical antigens have been described. Key features seem to be the occurrence of vimentin in the majority of mesotheliomas [23,24], a marker for mesenchymal cells and mesothelium, wide-spectrum cytokeratin [25-27] as a marker for epithelial cells and mesothelium, and positivity for podoplanin [28-31], which is a marker for lymphatic endothelium. These markers were also observed in mesotheliomas of rats [32].

In the present study, the tumor material was investigated histopathologically and immunohistochemically for the three markers cytokeratin, vimentin, and podoplanin.

All MWCNTs tested in this study showed a strong carcinogenic effect. Besides the diameter and length of MWCNTs, curvature seems to have had an influence on carcinogenic potency.

## Results

### Mortality

**Figure 1:**
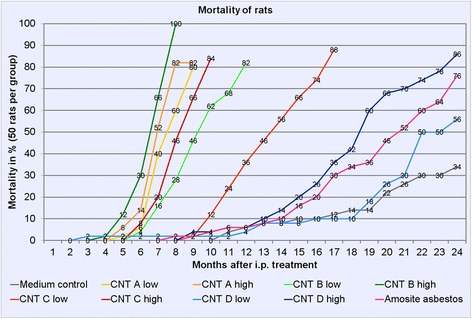
**Mortality curves in % of all rats of the study comprising all animals which died or were humanely killed due to a bad clinical condition.** A group was terminated when 80% mortality was reached.

In the MWCNT A and B low- and high-dose groups and in the MWCNT C high-dose group, 80% of the animals died or were humanely killed due to a bad clinical condition (for mortality curves see Figure 1). In the MWCNT C low-dose group, mortality reached 80% 16 months after the intraperitoneal treatment. The MWCNT D high-dose group showed 80% mortality after 23 months. At the end of the experimental time after 24 months, mortality was 56% in the MWCNT D low-dose group, 76% in the amosite asbestos group, and 34% in the negative control group.

### Macroscopic findings

At necropsy most animals of the MWCNT treatment groups and the majority of rats of the amosite asbestos group revealed ascites consisting of blood-containing fluid. Most animals showed adhesions and multifocal to coalescing small nodular lesions on the surface of the abdominal organs. Many rats had large nodules or tissue masses especially in the area of the diaphragm, liver, and pancreas, but the small intestine was also frequently involved. In some animals, we found aggregates of foreign material covered by a thin layer of serosa, primarily at the diaphragm and liver. The liver and spleen capsules were thickened and firm in numerous rats.

### Histopathological findings

#### Tumor incidences and time dependence

The most frequent tumors in the MWCNT and amosite asbestos treatment groups were malignant mesotheliomas of the peritoneum. In most cases these tumors were the obvious reason for a morbid condition of the rats and triggered the decision to perform euthanasia. In addition, we found a few pancreatic islet-cell adenomas, Leydig cell adenomas of the testes, and one renal lipoma. Tumors in other organs were infrequently and their incidences corresponded to normal spontaneous background.

**Figure 2:**
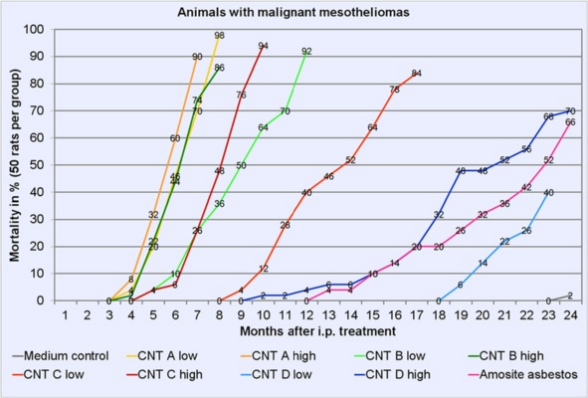
**Animals with malignant mesotheliomas over time in %.** A group was terminated when 80% mortality was reached.

Table 1 shows the incidences of malignant mesotheliomas including survival times of mesothelioma-bearing rats. The development of malignant mesotheliomas over time is shown in Figure 2.

**Table 1:** **Incidences of malignant mesotheliomas, mean survival times of morbid mesothelioma-bearing rats, and first day of mesothelioma detection**

**Substance**	Length (μm) WHO fibers*	Diameter (μm) WHO fibers*	Dose WHO fibers* 10 ^**9**^ **per rat (actual** ^**##**^ **)**	**No. of rats**	**Rats with mesothelioma**	**Mean survival time of mesothelioma-bearing rats (days)**	**Month of first detection of a morbid mesothelioma-bearing rat**	**Final month** ^**^
Medium control	-	-	-	50	1 (2%)	739	24	24
MWCNT A low	8.57 ± 1.51	0.085 ± 1.60	0.48	50	49 (98%)	213	5	9
MWCNT A high			2.39	50	45 (90%)	194	5	8
MWCNT B low	9.30 ± 1.63	0.062 ± 1.71	0.96	50	46 (92%)	294	6	13
MWCNT B high			4.80	50	45 (90%)	207	5	9
MWCNT C low	10.24 ± 1.64	0.040 ± 1.57	0.87	50	42 (84%)	415	10	18
MWCNT C high			4.36	50	47 (94%)	265	6	11
MWCNT D low	7.91 ± 1.40	0.037 ± 1.45	1.51	50	20 (40%)	666	20	24
MWCNT D high			7.54	50	35 (70%)	585	11	24
Long amosite asbestos	13.95 ± 2.10	0.394 ± 1.83	0.14	50	33 (66%)	623	14	24

*WHO fibers: length greater than 5 μm, diameter less than 3 μm, and a length-to-width ratio (aspect ratio) of at least 3:1.

^##^Actual WHO fiber number dose; deviations from target values are due to inhomogeneities during successive synthesis or suspension of test items.

^**^Last month when a mesothelioma-bearing rat was found in a group.

Rats in the MWCNT A groups developed a mesothelioma rate of 98% in the high-dose group and of 90% in the low-dose group. Mesothelioma-bearing animals had a mean survival time of 194 days in the high-dose group and of 231 days in the low-dose group. Mesotheliomas were detected in rats euthanized between 5 and 9 months in the low-dose group and between 5 and 8 months in the high-dose group.

In the MWCNT B groups, we found mesothelioma rates of 90% (high dose) and 92% (low dose). Survival times of mesothelioma-bearing rats were 207 days (high dose) and 294 days (low dose). Mesothelioma-bearing rats were euthanized between 5 and 12 months in the low-dose group and between 5 and 9 months in the high-dose group.

Incidences of mesotheliomas in the MWCNT C groups were 94% after treatment with the high dose and 84% with the low dose. In these groups, mesothelioma-bearing rats had a mean survival time of 265 days with the high dose and of 415 days with the low dose. Animals with mesotheliomas were observed between 10 and 18 months after treatment in the low-dose group and between 5 and 10 months after treatment in the high-dose group.

Animals of the MWCNT D treatment groups developed malignant mesotheliomas in 70% (high dose) and 40% (low dose). Survival times of the mesothelioma-bearing rats were 585 days with the high dose and 666 days with the low dose. We detected mesotheliomas in rats euthanized between 20 and 24 months in the low-dose group and between 11 and 24 months in the high-dose group.

In the amosite asbestos group, the mesothelioma incidence was 66%, with a mean survival time of 623 days. We observed mesotheliomas in rats euthanized between 14 and 24 months after intraperitoneal injection.

In the rats of the medium control group, we found one mesothelioma (2%) during final necropsies.

#### Other non-neoplastic MWCNT-induced histopathological findings in the abdominal cavity

**Figure 3:**
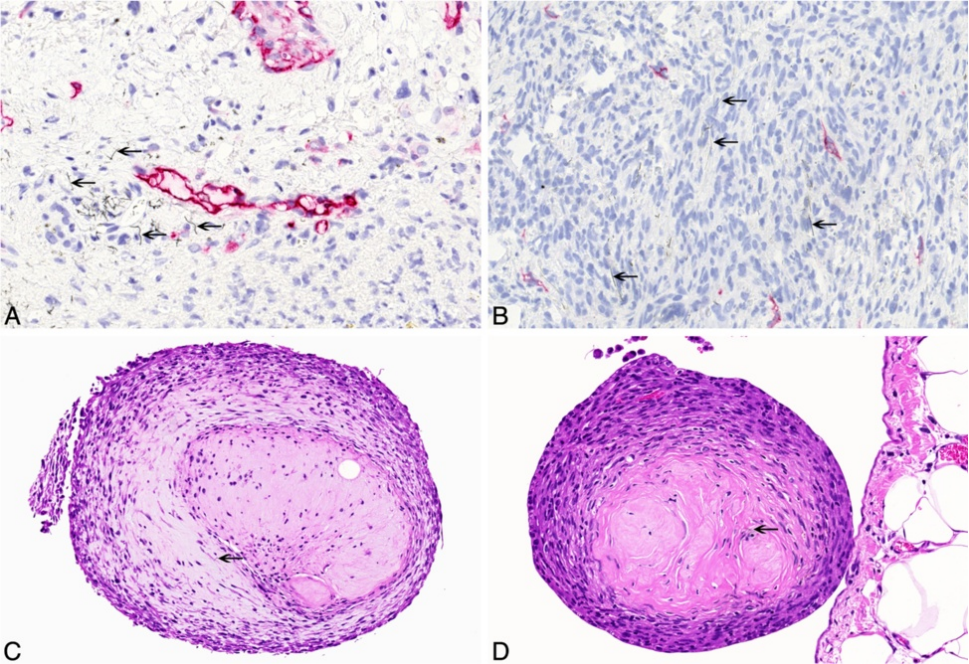
**Non-neoplastic histopathological findings in the abdominal cavity. A**: High-power view of anti-podoplanin immunohistochemistry showing single MWCNT A (high dose) nanotubes in the tissue (arrows). **B**: High-power view of anti-podoplanin immunohistochemistry showing single asbestos fibers in the tissue (arrows). **C**: H & E, high-power view of granuloma induced by MWCNT A (low dose) nanotubes including single nanotube (arrow, 25×). **D**: H & E, high-power view of granuloma induced by asbestos including single fiber (arrow, 40×).

Most of the MWCNT-treated rats showed multiple granulomas on the peritoneal surface of the abdominal organs and diaphragm. The granulomas frequently consisted of a central hyaline area which mostly included a few single fibers, surrounded by macrophages and lymphocytes. An outer layer of fibrocytes and collagen, covered by serosa, was mostly visible (see Figure 3). Organs such as the liver and spleen were engulfed by a thick layer of collagen-rich connective tissue intermingled with granulomas. In many cases, the liver showed adhesions to the diaphragm by fibrotic if not tumor tissue. We found agglomerates of foreign material underneath the serosa in many animals. Multifocal papillary projections of mesothelial hyperplasia were observed especially on the diaphragm and diaphragmatic liver surface.

Large aggregates occurred infrequently, whereas we observed single nanotubes in every slide (see Figure 3). Similar lesions were found in asbestos-treated animals (see Figure 3).

#### Histologic appearance of malignant mesotheliomas

Malignant mesotheliomas showed predominantly superficial growth on the serosal surface of the peritoneum of the liver, spleen, intestine, and abdominal wall. We frequently observed invasion into the mesentery, pancreatic tissue, and diaphragm. All in all, 320 of the 329 MWCNT-related malignant mesotheliomas had invaded the diaphragm, and 174 of these tumors showed invasion of the thoracic cavity. In the amosite asbestos group, which displayed 33 (66%) mesotheliomas, 29 mesotheliomas had invaded the diaphragm, and 5 of these tumors showed invasion of the thoracic cavity.

In the majority of cases, the liver was completely encapsulated, but it was frequently invaded as well. In contrast, invasion of the spleen along the vascular blood supply was rare. All malignant mesotheliomas showed some cellular pleomorphism; some of the tumors exhibited partly anaplastic round cells, especially those occurring early in the study. A couple of mesotheliomas developed areas with mucus production, others showed microcystic areas.

**Figure 4:**
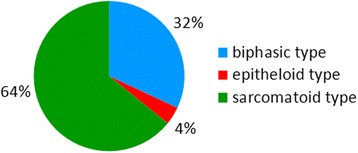
**Histological typing of malignant mesotheliomas occurring in the study.** The most frequent type of mesothelioma was the sarcomatoid type, followed by mesotheliomas of the biphasic type. Mesotheliomas of the epithelioid type were rare.

Based on their appearance in the H & E stain, we differentiated the malignant mesotheliomas into epithelioid, sarcomatoid, and biphasic types (see Figure 4). Malignant mesotheliomas of the epithelioid type occurred occasionally in the MWCNT groups B high, C low and high, D low and high, and in the amosite asbestos group. The malignant mesothelioma found in the medium control group displayed an epithelioid appearance. Epithelioid-type malignant mesotheliomas showed papillary structures or poorly formed glandular structures and round nuclei, and they infiltrated the adjacent connective tissue. All epithelioid-type malignant mesotheliomas were immunohistochemically positive for the markers pan-cytokeratin, podoplanin, and vimentin.

**Figure 5:**
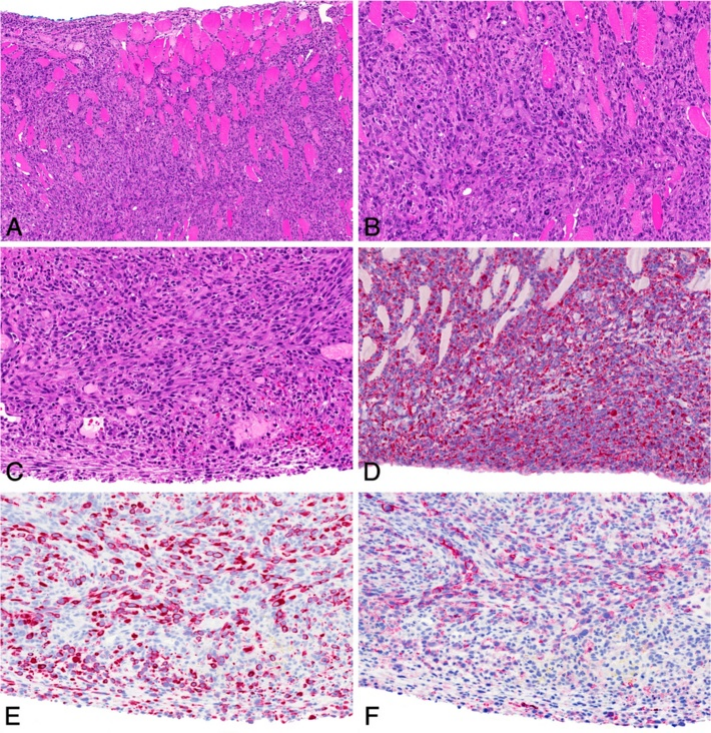
**Sarcomatoid-type malignant mesothelioma observed in the MWCNT B high-dose group. A**: H & E, low magnification of tumor tissue invading the diaphragm (20×). **B** and **C**: H & E, high-power view (40×). **D**: High-power view of anti-vimentin immunohistochemistry showing a strongly positive result (40×). **E**: High-power view of anti-pan-cytokeratin immunohistochemistry showing several positive cells (40×). **F**: High-power view of anti-podoplanin immunohistochemistry revealing positively stained cytoplasm of many cells (40×).

Malignant mesotheliomas of the sarcomatoid type (see Figure 5) were frequent in all MWCNT and amosite asbestos groups. The sarcomatoid-type mesotheliomas consisted of spindle-shaped cells with elongated nuclei forming bundles or whorls of cells. We observed osseous differentiation in a number of cases in each group. All sarcomatoid-type malignant mesotheliomas were immunohistochemically positive for the markers vimentin and podoplanin.

**Figure 6:**
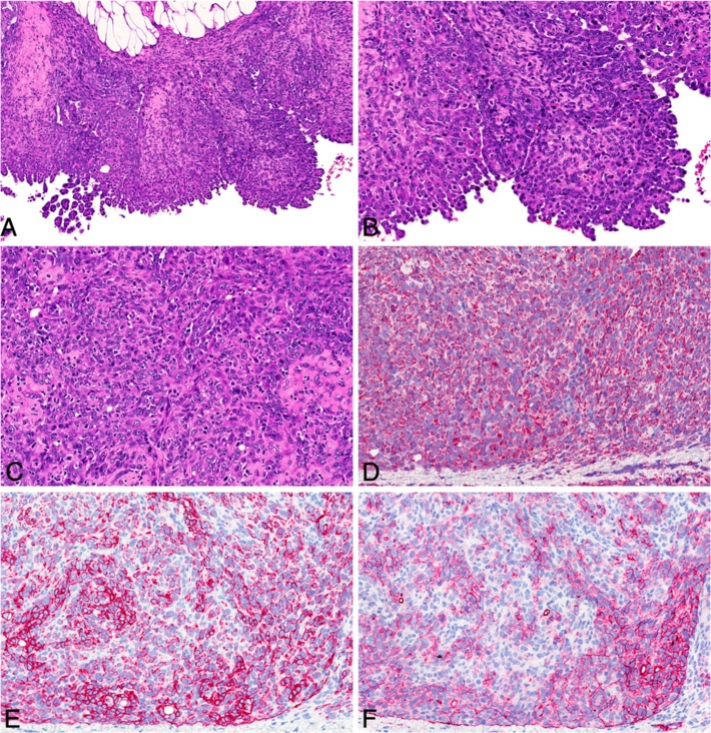
**Biphasic-type malignant mesothelioma observed in the MWCNT D low-dose group. A**: H & E, low magnification of tumor tissue found on the surface of the omentum (20×). **B** and **C**: H & E, high-power view (40×). **D**: High-power view of anti-vimentin immunohistochemistry showing a strongly positive result (40×). **E**: High-power view of anti-pan-cytokeratin immunohistochemistry showing positive staining of the majority of cells (40×). **F**: High-power view of anti-podoplanin immunohistochemistry revealing positively stained cytoplasm of many cells (40×).

**Figure 7:**
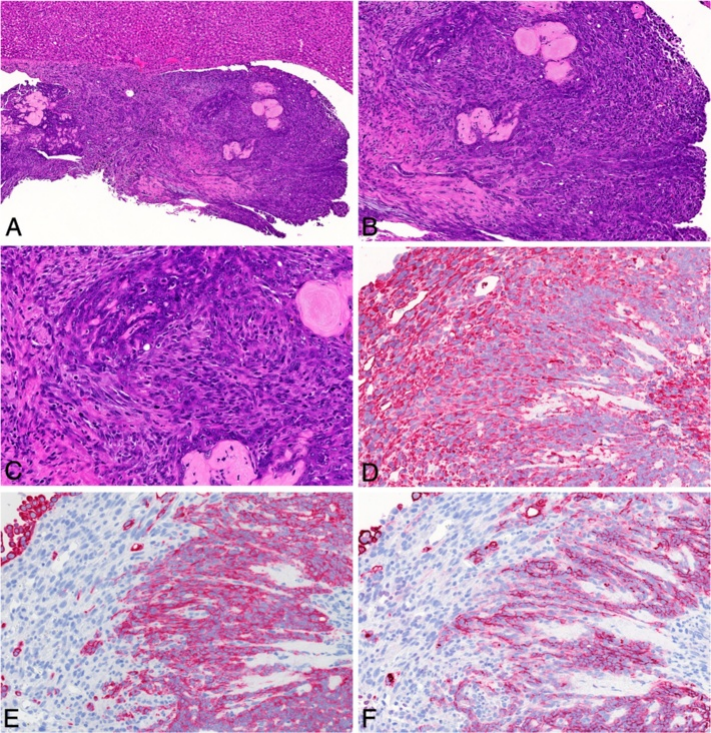
**Biphasic-type malignant mesothelioma observed in the amosite asbestos group. A**: H & E, low magnification of tumor tissue found on the liver surface (10×). **B**: H & E, higher magnification (20×). **C**: H & E, high-power view (40×). **D**: High-power view of anti-vimentin immunohistochemistry showing a strongly positive result (40×). **E**: High-power view of anti-pan-cytokeratin immunohistochemistry showing positive staining of many cells (40×). **F**: High-power view of anti-podoplanin immunohistochemistry revealing positivity in distinct parts of the tumor.

Malignant mesotheliomas of the biphasic type, comprising both epithelioid and sarcomatoid patterns, were also seen in animals of all MWCNT groups (see Figure 6) and the amosite asbestos group (see Figure 7). Several of these tumors exhibited osseous differentiation in parts of the lesion. All biphasic-type malignant mesotheliomas were immunohistochemically positive for the markers vimentin, podoplanin, and pan-cytokeratin.

Mesotheliomas occurring in the early phase of the study more frequently showed a higher number of poorly differentiated cells or areas of anaplastic cells.

Malignant mesotheliomas in the amosite asbestos group developed the same histological appearances and reactions to immunohistological markers as the MWCNT-induced mesotheliomas.

Overall, the study revealed highest incidences for the sarcomatoid type of mesothelioma, followed by the biphasic type. Mesotheliomas of the epithelioid type were rare in our study.

## Discussion

The hypothesis of a mesothelioma-inducing potential of multi-walled carbon nanotubes, assumed because of their fiber-like dimensions and biopersistence comparable to asbestos fibers, was confirmed by our experimental investigations with rats treated intraperitoneally with tailor-made MWCNTs which were suspended in fluid according to Porter and co-workers [17] similar to surfactant-containing lung fluid. Amosite asbestos fibers served as positive control. A dose-dependent difference in the carcinogenic response, however, is difficult to deduce from our study. Tumor incidences in all MWCNT dose groups treated with 10^9^ or 5 × 10^9^ nanotubes were 40 to 100%. Our experimental results thus clearly provide proof of the mesothelioma-inducing potential of these MWCNTs in rats. However, the data cannot be used for risk assessment. This is especially true because we have no data on the carcinogenic potential of MWCNTs in rats after inhalation exposure and in particular also no information on the exposure concentration needed to reach a sufficient dose of MWCNTs at the pleura to induce mesotheliomas.

As positive control, we injected a long type of amosite asbestos, dosed at a concentration of 1 × 10^8^ WHO fibers per animal. Using this dose, a mesothelioma incidence of approximately 50% could be expected. Davis and co-workers [16], who investigated the UICC standard reference of amosite asbestos, reported the following data: At doses of 0.5, 2.5 mg, and 15 mg amosite, corresponding to 1.7 × 10^7^, 8.5 × 10^7^, and 5.1 × 10^8^ fibers with a length greater than 5 μm, respectively, they found mesothelioma incidences of 46.9%, 59.4%, and 79.2%. At a dose of 1 × 10^8^ fibers greater than 5 μm, a mesothelioma incidence of approximately 50% can be predicted [16]. In the present study, the incidence of mesotheliomas caused by asbestos was 66%.

There are various hypotheses on the mechanisms that might contribute to the induction of tumors by biopersistent fibrous particles. The carcinogenic effect of the tested nanotubes might be due to frustrated phagocytosis followed by chronic inflammation, which has been described for asbestos fibers [33,34] and for MWCNTs as well [35]. An exaggerated inflammatory response of the peritoneal mesothelium with granuloma formation was observed by Poland and co-workers [12] after injection of long but not short amosite asbestos fibers and also after injection of MWCNTs with a large proportion of long fibers. Poorly soluble but otherwise non-toxic particles that are non-digestible and non-degradable can harm macrophages when they take up a large volume of this material or when this material activates and damages the macrophages due to its long fiber shape. These macrophages will no longer be able to move properly and eliminate the foreign material. Activated and apoptotic macrophages and interaction of the foreign material with surrounding epithelial cells then causes an inflammation that becomes chronic because of the continuing irritant and damaging effect of the foreign material. This particle-induced chronic inflammation and the high concentration of reactive oxygen and nitrogen species generated in the course of the inflammatory process can also lead to oxidative damage and genotoxic effects. A significant increase in reactive oxygen species was measured in monocyte-derived macrophage monocultures after administration of surfactant pre-coated MWCNTs, whereas uncoated MWCNTs caused only a minor reaction [36]. In our study, the carbon nanotubes tested were suspended in a fluid [17] containing 1,2-dipalmitoyl-sn-glycero-3-phosphocholine (DPPC), a major component of surfactant. We used this fluid for dispersion, because it prevented agglomeration of the MWCNTs during dosing of the animals. The surface of the nanotubes in our study was otherwise free of chemicals and unloaded, so that they were hydrophobic. Functionalization of carbon nanotubes affects their bioactivity and changes surface reactivity, surface loading, stability, dispersability, and agglomeration [37]. A short-term *in-vivo* test [38] demonstrated a different behavior of functionalized MWCNTs, interpreted as an influence on nanotube size and agglomeration. Our non-functionalized hydrophobic MWCNTs dispersed well in the DPPC-containing medium, avoiding aggregation of nanotubes. Aggregated nanotubes would have produced a different reaction because of the resulting change in bioavailability, width, length, surface area, shape, and other factors. The hydrophobicity of carbon nanotubes enables them to penetrate cell membranes quite easily. Penetration of uncoated MWCNTs with a diameter of 49 nm and a length of 3.86 μm into lung epithelial cells has been demonstrated [39].

Furthermore, uncontrolled cell proliferation may be the result of CNT interaction with the mitotic spindle apparatus, which has been described *in vitro* for asbestos fibers [40], for 1- to 4-nm-thin single-walled CNTs [41], and for 10- to 20-nm-thin MWCNT fibers [42]. The impact of MWCNT treatment on the cell cycle resulted in an increase in cells in the proliferation phase. The MWCNTs in our study were more than twice the diameter of the nanotubes used by Siegrist and co-workers [42], therefore, interaction with the mitotic spindle cell apparatus may be less likely.

The results of our study are in contrast to the findings by Varga and Szendi [43], who found induction of granulomas but not mesotheliomas in Fischer 344 rats. They used single-walled CNTs with a diameter of less than 2 nm and lengths of 4 to 15 μm and short multi-walled CNTs with diameters of 10 to 30 nm and lengths of 1 to 2 μm. Furthermore, their experimental time was only one year. Their negative finding in respect of tumors after treatment with MWCNTs may be due to the short nanotube length of only 1 to 2 μm. At least, the carcinogenic potential of mineral fibers with a length below 5 μm was questioned. The single-walled CNTs were less than 2 nm in diameter, leading presumably to a curled shape getting tangled, resulting in the occurrence of almost no single CNT but rather a particle-like appearance and behavior. Another difference from our study was the mode of administration. Varga and Szendi implanted the CNT fibers, encapsulated in hard gelatin capsules, into the Kertai fold located on the inner liver surface, probably leading to reduced deposition of nanotubes at the serosa. In our study, we injected the fibers directly into the abdominal cavity, suspended in a fluid.

No mesotheliomas were observed after intraperitoneal injection of multi-walled carbon nanotubes measuring 11 nm in diameter and with a length of most nanotubes below 5 μm into Wistar rats [14]. Nanotubes with diameters of less than 20 nm most likely have a curled shape and are tangled, and together with the relatively short length of most fibers this is responsible for the negative outcome in respect of tumors. The thinnest nanotubes used in our study, which had more than twice the width of the nanotubes tested by Muller and co-workers [14], showed a later onset of tumor morbidity compared with our other MWCNTs, but the induced tumor incidence was still quite high.

In another study, MWCNTs were injected subcutaneously in rasH2 mice [44]. The authors implanted the test material, which had a mean diameter of 100 nm and a mean length of 10 micrometers, into the subcutaneous tissue. They evaluated the tumor outcome after 26 weeks, but observed neither tumors nor inflammatory cells at the injection site. There are no data in the literature showing tumors after subcutaneous injection of mineral fibers, except the report about mesothelioma induction in mice by subcutaneous injection of asbestos fibers by Roe and co-workers [45], and even for a tumor response based on a foreign-body effect the experimental time was too short to allow final conclusions to be drawn.

Mitsui MWCNT-7 carbon nanotubes with an average width of 100 nm and a length of 1 to 20 μm (median of 2 μm) administered by intraperitoneal injection into heterozygous p53 mice [13,46] and by intrascrotal injection into Fischer 344 rats [47] caused mesotheliomas as well. Mitsui MWCNT-7 show a needle-like straight structure, probably due to their relatively large diameter, and this kind of MWCNTs is structurally very similar to carcinogenic mineral fibers. This is in accordance with our observations on our tailor-made MWCNTs. The more needle-like MWCNTs A and B with mean widths of 85 and 62 nm, respectively, seemed to have a stronger carcinogenic potency than MWCNTs of type C with a diameter of 40 nm and a more bent structure than MWCNTs A and B. At least the low-dose MWCNT C group showed a slightly later onset of tumor-related morbidity compared with MWCNTs A and B. The later onset of tumor-related morbidity and moderate mesothelioma incidences in the MWCNT D groups may be due to the same reason. In the scanning electron microscopy photographs this type of nanotube shows a more curved shape compared with MWCNTs A, B, and also C. The bent nanotube material D had the highest portion of WHO fibers with a length greater than 5 μm, but the lowest portion of WHO fibers with a length greater than 20 μm. It is known that the carcinogenic potency of biopersistent mineral fibers increases with growing fiber length. Frustrated phagocytosis and long clearance half-times in the pleural space may play a role in this context [3].There must be other factors than length and diameter influencing the effect of MWCNTs. The kinetic behavior of bent MWCNTs is most probably different from that of straight MWCNTs, which might influence clearance half-times.

Nagai and co-workers [15] demonstrated cytotoxicity of thin and rigid MT50a-MWCNTs with a diameter of 50 nm and a length of 5.29 μm *in vitro*. In contrast, thick NT115- and NT145-MWCNTs with a diameter of 150 nm and a length of 4.88 or 4.34 μm, respectively, and tangled NTtngl nanotubes with diameters of 2 to 20 nm and a length of 3 μm were less toxic.

The same authors observed mesotheliomas in rats after i.p. injection of MWCNTs. The diameters of these nanotubes were 50, 116, and 143 nm, but their average length was only between 4 and 5 μm. The study report provided no information as to whether the various types of nanotubes used differed in shape (straight, needle-like, or rather curved or wavy). The authors treated rats with 10^7^ to 10^8^ nanotubes per animal and observed the effects for one year. The number of rats available for histopathological investigations was quite different per experimental group, ranging from 6 to 43. Incidences of mesotheliomas observed per group were mostly quite high, reaching 80 to 100%. Nanotubes of 50 nm in diameter caused more tumor-bearing animals after one year than nanotubes of 143 nm diameter after treatment of the rats with identical number concentrations of nanotubes. Based on this result, the authors suggested reduced carcinogenic potency for nanotubes with lower diameters. No tumors were found in 6 rats treated with tangled nanotubes with a diameter of 15 nm, even after up to three years of observation time [48]. No tumors were found either in 15 rats treated with the same tangled nanotubes after one year of experimental time. The average length of the nanotubes used in this experiment was quite low, but the frequency distribution in the lengths of the various nanotubes used showed some differences, especially in the length range of 7 to 13 μm. We think that a major effect of the length of nanotubes on their carcinogenic potency cannot be ruled out. Tangled nanotubes behave like granular particles, with almost no single nanotubes available to induce mesotheliomas.

The observations by Nagai and co-workers [15] were in concordance with the results of our study, where all MWCNTs with mean diameters between 37 and 85 μm and mean lengths between 8 and 10 μm (the length distribution within each CNT sample showed a certain percentage of CNTs with lengths greater than 20 μm) showed a carcinogenic effect. In our study, the more bent but not tangled or agglomerated MWCNTs D had a longer latency period and thus lower carcinogenic potency in comparison with the more straight and needle-like MWCNTs A, B, and C. The absence of mesotheliomas after three years in rats treated with tangled MWCNTs [15,48] is in line with the hypothesis that very thin, and curled and tangled CNTs only occur as agglomerates and their toxic effects are very similar to the ones of biopersistent granular particles. Pauluhn and co-workers [49] described such particle-like toxic effects in a subchronic rat inhalation study with Baytubes®. These nanotubes are 10 to 15 nm in diameter and 2 to 10 μm in length. They were very curled and tangled, occurring only as agglomerates, and therefore the typical particle-like effects in rat lungs including alveolar macrophage overload but no fiber or single CNT-related effects occurred. Similar observations were made by Ma-Hock and co-workers [50], who tested Graphistrength™ MWCNTs with diameters of 10 to 15 nm and lengths of 1 to 10 μm in a short-term rat inhalation study (6 days with a 3-week post-inhalation period). These carbon nanotubes were agglomerated in bundles and caused granuloma formation in the lungs. A carcinogenic potential of such tangled and agglomerated nanotubes at low doses seems to be unlikely, because phagocytosis by alveolar macrophages is given. For the pleural cavity, Donaldson and co-workers [3] assumed a risk of asbestos-like pathogenicity for nanotubes that are longer than 5 μm. Longer fibers cannot negotiate the stomata (2–12 μm) and are retained there, so that they can interact with the mesothelium and immune cells.

Nagai and co-workers [15] also performed rat genome microarray CGH analysis of mesothelioma samples from their MWCNT groups. In the mesothelioma samples, whose signal intensity at the Cdkn2a/2b locus was largely lower than −1, they found homozygous deletion of the gene. Furthermore, they detected deletion of this gene locus also in their asbestos-induced mesothelioma samples.

The mechanism of mesothelioma induction by the tested MWCNTs seemed to be very similar to those triggered by asbestos. A known factor contributing to tumor induction by some mineral fibers is the presence of transition metals which may cause metal-catalyzed free radical chain reactions, as in the case of iron and hydroxyl radical formation via the Fenton reaction. The nanotubes applied in our study were generated using iron or cobalt as catalysts, which were incorporated into the core of the nanotubes. We did not detect dissolution of these metals, however, we also cannot completely exclude that some not measurable metal components were involved in the toxic effects, although this seems very unlikely in our opinion. The possibility that insoluble metals included on the particle surface could cause an immune reaction was discussed formerly [51]. X-ray photoelectron spectroscopy, however, revealed no metals on the surface of the MWCNTs used in the present study.

Since biopersistent granular dusts are not able to induce mesotheliomas after intraperitoneal injection, even at very high doses of 4 × 20 mg per rat i.p. [9], it is very unlikely that the mass doses administered in the present study, ranging from 0.05 to 3.0 mg per rat, induced the mesotheliomas per se.

To explain the carcinogenic potential of the MWCNTs used in our study, we would primarily point to the high numbers of nanotubes fulfilling the criteria for WHO fibers in combination with their lack of entanglement and of agglomeration in the suspension, which is due to their thickness and the resulting needle-like shape. The rigidness of the different multi-walled carbon nanotubes is difficult to estimate and also dependent on their thickness. A nanotube length of more than 15 to 20 micrometers, preventing clearance from the serosal cavities because of limited stoma size and leading to frustrated phagocytosis and subsequent chronic inflammation, seems to be one mechanism of mesothelioma induction, however, it is probably not the only pathway. The mean length of our four MWCNTs was below 12 micrometers, which is the maximum size of stomatal openings in rats [52], but the test material also included a certain percentage of nanotubes longer than 20 μm.

In 97% of the MWCNT-treated mesothelioma-bearing rats, the diaphragm was invaded by tumor cells, whereas the serosas of the lateral and ventral abdominal walls were only incidentally involved. The high occurrence of mesotheliomas at this location is most probably due to the fluid flow in the peritoneal cavity towards the diaphragm. The suspended MWCNTs were injected into the inguinal region, but the majority of tumor tissue was found at the diaphragm and adjacent tissue structures. The location of induced tumors can be explained by the drainage flow of peritoneal fluid towards stomata and subsequently into lymphatic vessels. In rats, the majority of lymphatic stomata are located at the diaphragm [53]. Further peritoneal stomata are present in the omentum, mesentery, pelvic peritoneum, anterior abdominal wall, and liver [54]. Therefore, larger amounts of carbon nanotubes may be transported towards the lymphatic stomata at the diaphragm. It is supposed that nanotubes may be transported to submesothelial tissue through the peritoneal stomata. Adjacent cuboidal mesothelial cells are connected by intercellular tight junctions, which limit migration of macrophages or transport of red blood cells, particles, or plasma proteins to the submesothelial tissue. For the pleural cavity, stomata are likewise the only connection to the lymphatics [55] and thus the main path for larger particles, fibers, and nanotubes to be eliminated by flow or after phagocytosis by migrating macrophages [56]. The stomata consist of an outer mesothelial margin and an inner lymphatic endothelial orifice [57], with round or oval openings [56]. Specifications of the size of lymphatic stomata in rats vary from 4.5 to 8 μm [58] to 4 to 10 μm in diameter [57]. For humans, stomata 2 to 12 μm in diameter have been reported [3]. Their size and shape may depend on the contractile state of the diaphragm at the time of fixation. The stomata may function as a sieve, which longer fibers or nanotubes may be unable to pass. Longer nanotubes or fibers like those used in our study may accumulate at this location, especially at the smaller stomata, and can impact into the adjacent mesothelium or plug the stoma. Very small particles or very short nanotubes may be incorporated with fluid into mesothelial cells by pinocytosis [57]. The primary unidirectional valves in the walls of the initial lymphatics allow and regulate entrance of serosal fluid into the lymphatic network preventing fluid backflow. The initial lymphatic vessels located in the submesothelial tissue have diameters of 1 to 10 μm [53]. The high number of longer nanotubes used in our study might have had great impact on the area of the diaphragmatic sieve and stomata openings. The increase in intraabdominal fluid in most rats was not only a result of increased production due to inflammatory processes, but might also have been a result of decreased transport capabilities of the stomata.

The peritoneal tumors observed in our study closely resemble human malignant mesothelioma with regard to biological behavior, histopathology, and immunohistochemical features. In our study, the histological features of the MWCNT-induced mesotheliomas and the reaction patterns of the immunohistochemical markers were congruent with those observed for amosite asbestos-induced mesotheliomas. The immunohistochemical results were also in accordance with the observations by Sargent and co-workers [59], who found serosal tumors after methylcholanthrene treatment and additional MWCNT inhalation in mice. The reaction patterns described by Blackshear and co-workers [60] for spontaneous mesotheliomas collected from Fischer rats are also in agreement with our observations.

Spontaneous mesotheliomas in rats occur at a low incidence. In aged Wistar rats, incidences are up to 4% in males and 0% in females [61]. In male Fischer 344 rats, mesotheliomas most frequently are restricted to the serosa of the tunica vaginalis, and they are rare [60]. In our medium control group, we found one mesothelioma (2%). Consequently, the mesotheliomas we found in the MWCNT groups of our study at incidences ranging from 40 to 98% must be regarded almost completely as induced by the tested MWCNTs.

There is thus a very high probability that a variety of multi-walled carbon nanotubes are also potent carcinogens like asbestos for humans. Our test was designed for hazard identification of MWCNTs, not for risk assessment. We were able to show that the selected carbon nanotubes had mechanistic potency to induce tumors, comparable to that of asbestos fibers. Inhalation exposure to lower doses of these carbon nanotubes should be performed in the future to enable proper risk assessment. The diameter, length, and shape of the carbon nanotubes seem to be essential parameters determining their carcinogenic potential.

## Conclusion

Because of their special mechanical, structural, chemical, and physical properties, carbon nanotubes have great economic relevance and production volumes are rapidly increasing. Production and processing of nanotubes and nanotube-containing materials are occupational situations where inhalation exposure to nanotubes may occur and a carcinogenic risk cannot be ruled out. Likewise, the staff of research labs handling nanotubes should also be aware of the potential carcinogenic risk, which is comparable to that of asbestos fibers. The question immediately arises whether all nanotubes behave in the same way, also in respect to their potential carcinogenic risk. A similar question had to be answered about three decades ago: do all mineral fibers have the same carcinogenic potential and potency and do they pose the same risk as asbestos fibers, which were proven to be carcinogenic in humans? All man-made mineral fibers longer than 5 μm and thinner than 3 μm and having a length-to-diameter ratio of at least 3:1 have to be treated as potential human carcinogens, if their half-time in rat lung is similar to the half-time of mineral fibers known to induce tumors in humans or rats after inhalation exposure. Or, putting it the other way round, mineral fibers with low biopersistence in rat lung and thus a half-time below a certain number of days can be excluded from the suspicion of being human carcinogens. Contrary to mineral fibers which, depending on their chemical composition, can have quite different solubility properties in lung or alveolar macrophages, all nanotubes are of high durability because of their composition of inorganic carbon only. Besides solubility, the half-time in the lung depends on the length of fibers or nanotubes and, in this respect, there seems to be no difference at a first glance between fibrous mineral or fibrous carbon particles. Based on this consideration and because of the high durability of carbon nanotubes, their clearance from the lung or pleural space seems to depend mostly on their length: the longer the carbon nanotubes, the longer their residence time in the target organ and the stronger their toxic effect. And yet, there may be a characteristic of carbon nanotubes that may modify their toxic and carcinogenic potency, and according to our preliminary data and hypothesis this could be the shape of nanotubes. The more bent, curved, or waved the different types of nanotubes, the lower their toxic and carcinogenic potency seems to be. Their kinetic behavior may be slower and cell interactions less intensive. In other words, carbon nanotubes having a straighter and more needle-like shape can be expected to display higher toxic and carcinogenic potency. In addition, comparable to mineral fibers, it seems that the longer the nanotubes or fibers, the stronger their toxic and carcinogenic potency. On the other hand, at the extreme, strongly curled and tangled carbon nanotubes that exist mostly as agglomerates are less likely to be potential contributors to fiber-specific toxicity.

## Methods

### Test material

For the carcinogenicity study, we used four different tailor-made multi-walled carbon nanotubes (see Table 2), produced by means of the chemical vapor deposition method. The fibers differed in length and diameter, but also to some extent in the production process in that it used diverging carbon sources or catalysts. Because of the high temperatures during synthesis (850–900°C for cyclohexane and acetonitrile and 1100–1150°C for benzene), the carbon nanotubes were free of organic chemical remnants on their surfaces. Traces of the catalyst were minimal. The MWCNT surfaces were uncharged and uncoated.

**Table 2:** **Dose groups, substances and dosing**

**Substance**	**Carbon source**	**Catalyst**	**Generation method**	**Length (μm) all fibers**	Length (μm) WHO fibers*	Diameter (μm) WHO fibers*	**Specific fiber concentration (10** ^**9**^ WHO fibers*/mg)	Fibers [length >20 μm] (% of WHO fibers*)	Dose WHO fibers* 10 ^**9**^ **per rat (targeted** ^**#**^ **)**	**Dose WHO fibers 10** ^**9**^ **per rat (actual** ^**##**^ **)**	**Dose (mg in 2 ml)** ^**###**^	**Inner angle**	**No. of rats**
Medium control	-	-	-	-	-	-	-	-	-	-	-	-	50
MWCNT A low	Benzene	Fe	Sublimation method	2.72 ± 2.29	8.57 ± 1.51	0.085 ± 1.60	2.39	3.81	1	0.48	0.2	174.85° ±5.82°	50
MWCNT A high									5	2.39	1.0		50
MWCNT B low	Cyclohexane	Fe	Aerosol method	2.13 ± 2.46	9.30 ± 1.63	0.062 ± 1.71	1.60	9.35	1	0.96	0.6	148.10° ±11.48°	50
MWCNT B high									5	4.80	3.0		50
MWCNT C low	Cyclohexane	Fe	Aerosol method	4.18 ± 2.41	10.24 ± 1.64	0.040 ± 1.57	10.91	11.77	1	0.87	0.08	144.55° ±11.48°	50
MWCNT C high									5	4.36	0.4		50
MWCNT D low	Acetonitrile	Co	Aerosol method	2.53 ± 2.02	7.91 ± 1.40	0.037 ± 1.45	30.15	2.13	1	1.51	0.05	Not measured	50
MWCNT D high									5	7.54	0.25		50
Long amosite asbestos	-	-	-	6.22 ± 3.12	13.95 ± 2.10	0.394 ± 1.83	0.1	28.55	0.1	0.14	1.4	-	50

Fiber types, lengths and diameters (geometric mean, GMSTD), % of WHO fibers* (length greater than 20 μm) and inner angles (to evaluate the degree of circumflexion of the different CNTs, the angle at the concave side [inner angle] was measured between two straight lines, the first from the beginning of the circumflexion to the middle and the second from the middle to the end of the circumflexion).

*WHO fibers: length greater than 5 μm, diameter less than 3 μm, and a length-to-width ratio (aspect ratio) of at least 3:1.

^#^Targeted fiber number dose according to WHO guideline and TRGS 905.

^##^Actual fiber number dose; deviations from target values are due to inhomogeneities during successive synthesis or suspension of test items.

^###^Total mass dose per rat in mg of substance including also fibers shorter than WHO fibers.

### Characterization of carbon nanotubes

Measuring the defect density by Raman spectroscopy revealed a ratio of the 1347/cm and 1580/cm peak of 1.0 to 1.3 for all four MWCNTs. Applying the Brunauer-Emmet-Teller (BET) method, the specific surface area of the different MWCNTs ranged between 52 and 60 m^2^/g. X-ray photoelectron spectroscopy of the MWCNT surface showed a carbon content of 94.72 to 99%. The remaining elements consisted of nitrogen (0.0 to 1.5%) and oxygen (1.0-4.93%), depending on the carbon source. While the acetonitrile source resulted in the highest nitrogen amount (1.5%), cyclohexane and benzene caused only very low amounts (<0.27%). The oxygen content also depended slightly on the carbon source, with benzene causing 1.0% and cyclohexane up to 4.93%.

MWCNTs A and B had a medium length of 8.57 μm and 9.30 μm, respectively. With 85 nm and 62 nm their diameters were a little thicker than those of MWCNTs C and D, which were 40 nm and 37 nm, respectively. Differences between MWCNTs A and B are the carbon source (A: benzene; B: cyclohexane) and the generation method (A: sublimation method; B: aerosol method). The catalyst for both fibers was ferrocene.

MWCNT C is a thin, long fiber (40 nm in diameter and 10.24 μm in length) produced by the aerosol method with cyclohexane as carbon source and ferrocene as catalyst.

MWCNT D is a thin fiber of medium length (37 nm in diameter and 7.91 μm in length). It was produced by the aerosol method with acetonitrile as carbon source and cobaltocene as catalyst.

Both MWCNTs C and D have nearly the same diameter, but MWCNT C has a greater length and a much higher proportion of WHO fibers that are longer than 20 μm.

Table 2 shows the mean diameters and mean lengths of our tailor-made MWCNTs A, B, C, D and of the amosite asbestos used.

### Characterization of amosite asbestos

The long amosite sample used as reference material was received from Johns Manville Corp. (Littleton, CO, USA) on August 25, 1997.

### Dispersion of nanotubes and fibers

For the intraperitoneal test, we performed dispersion of carbon nanotubes and amosite asbestos fibers in “artificial lung medium” according to Porter and co-workers [17]. Our dispersion medium consisted of Ca^2+^- and Mg^2+^-free phosphate-buffered saline, pH 7.4 (Biochrom AG, Berlin, Germany), supplemented with 5.5 mM D-glucose, 0.6 mg/ml bovine serum albumin (Sigma-Aldrich, Seelze, Germany), and 0.01 mg/ml 1,2-dipalmitoyl-sn-glycero-3-phosphocholine (Sigma-Aldrich, Seelze, Germany). To create the suspensions we used an ultrasonic homogenizer (Bandelin, Sonoplus HD 2070, VS 70 T) twice for 5 minutes (90% duty cycle, amplitude of 80 μm/ss).

**Figure 8:**
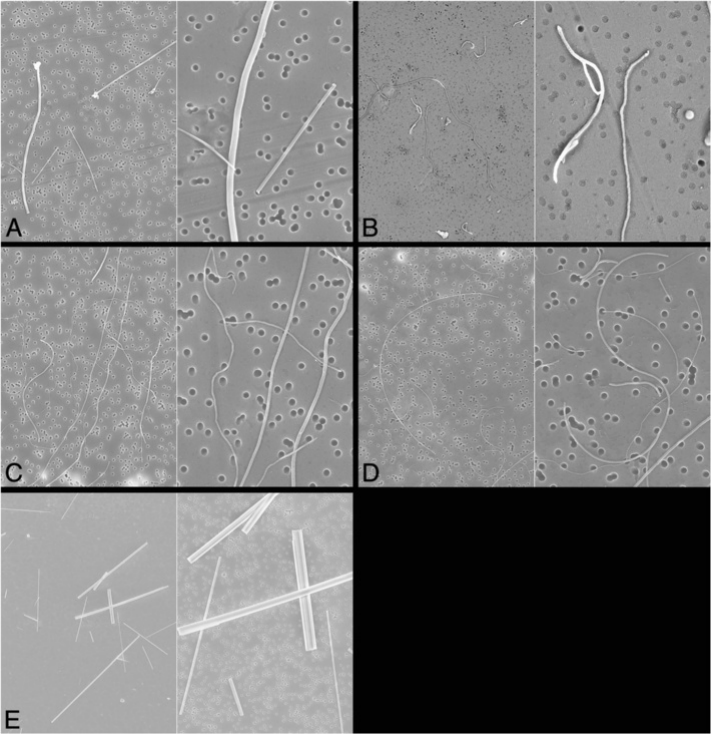
**Scanning electron microscopic photographs of carbon nanotubes and asbestos fibers in suspension.** Photographs were taken from liquid formulations of CNT in medium according to Porter (physico-chemical characterization in pre-trials). CNT agglomerates occurred infrequently and are not visible here. **A**: MWCNT A (left: 10,000×, right: 30,000×). **B**: MWCNT B (left: 10,000×, right: 30,000×). **C**: MWCNT C (left: 10,000×, right: 30,000×). **D**: MWCNT D (left: 10,000×, right: 30,000×). **E**: Long amosite asbestos (left: 2,000×, right: 7,000×).

Successful homogenization of the suspended nanotubes was proven by SEM. Their length and diameter distributions were measured again (see Figure 8A-E). The SEM images shown were prepared from the suspensions of the respective last pre-test for nanotube dispersion. From each suspension we transferred a very small volume of fluid to the SEM mesh. We cannot exclude that some aggregates may be in the major parts of the suspension that were not analyzed. The SEM images reflect the same fiber number concentrations as used for intraperitoneal injections.

### Dosing scheme

Treatment (see Table 2) consisted in a single intraperitoneal (i.p.) injection of the test substance in the inguinal region. We selected the target fiber number concentrations of 1 × 10^9^ and 5 × 10^9^ WHO fibers [1] per animal according to the EU protocol [7] and the German guideline TRGS 905 [62] for carcinogenic, mutagenic, and reproduction hazardous materials. WHO fibers are defined as fibers with a length greater than 5 μm and a diameter less than 3 μm and a length-to-width ratio (aspect ratio) of at least 3:1 [7]. As the test items had to be produced successively in aliquots, deviations from the initially determined values were found due to inhomogeneities in the final test item bulks. Thus, the actual doses showed some deviation from the targeted doses. Table 2 shows the actual mass doses per animal. The study duration was scheduled for 24 months after i.p. injection.

The positive control group was treated with amosite asbestos at a ten-fold lower number concentration. We determined this concentration for i.p. administration of amosite asbestos based on published data showing that 1 × 10^8^ WHO amosite asbestos fibers will cause about 50% mesothelioma-bearing rats after two years of experimental time [16].

### Animal experiment

The study was approved according to the German Animal Welfare Act by the local authority at the LAVES Niedersachsen, Hannover, Germany, No. 33.9-42502-04-11/0507; 33.9-42502-04-11/0743.

For the carcinogenicity test we used 500 male Wistar Han rats Crl:WI(Han), 50 for each group (Charles River Deutschland, Sulzfeld, Germany).

All rats were housed in groups of four rats in polycarbonate cages (Macrolon® type IV) on absorbent softwood bedding (Lignocell BK 8–15, JRS GmbH & Co KG, Rosenberg, Germany). Tap water (Stadtwerke Hannover, Germany) and pelleted food (R/M-H V1534, Ssniff, Soest, Germany) was offered *ad libitum* and refreshed at least once weekly. Standard animal housing conditions comprised a temperature of 20 ± 2°C and a 12-hour light/dark cycle.

For unequivocal animal identification, the rats were tattooed at the ears. Animals were checked at least once daily for clinical symptoms, morbidity, or mortality. Body weight recordings were performed at weekly intervals.

Rats were kept for 24 months after i.p. treatment and sacrificed at the end of the study or previously euthanized to avoid unnecessary pain and suffering if they were found morbid and were expected to die soon. All animals which were humanely killed due to a bad clinical condition or which died were included in the mortality curves.

### Histology

We performed complete necropsy on all animals that were euthanized either because they were in a bad clinical condition or because a mortality of 80% was reached in the corresponding group, or at the end of the study. Organs and pathologic lesions were fixed in 10% buffered formalin for 24 hours. If subsequent trimming was not possible due to a weekend, the tissue material was transferred to 70% ethanol for further fixation up to 48 hours. All macroscopic lesions, pre-defined parts of diaphragm, abdominal wall, liver, spleen, kidney, mesentery, intestine, lung, and brain were embedded in paraffin. Three-μm-thick sections were prepared and routinely stained with hematoxylin-eosin.

We used a bright-field microscope for evaluation of the slides. Tumors and pre-neoplastic lesions of the mesothelium were classified according to the INHAND (International Harmonization of Nomenclature and Diagnostic Criteria for Lesions in Rats and Mice) nomenclature [22]. A second certified veterinary pathologist reviewed tumors and borderline lesions.

### Immunohistochemical analysis

From each of the dose groups, we selected ten wax blocks with tumor tissue for immunohistochemistry. A selection factor was the duration of fixation, which we tried to keep as short as possible.

For immunohistochemical detection of the chosen markers for tumor tissue, we cut 3-μm-thick paraffin sections from the histology wax blocks containing the tumor material and mounted them on glass slides. Paraffin sections were then dewaxed and subject to antigen retrieval methods validated for each of the primary antibodies.

The primary antibodies used comprised monoclonal mouse anti-vimentin (clone V9, isotype IgG1 kappa, Dako Denmark A/S, 2600 Glostrup, Denmark, #M 0725), monoclonal mouse-anti-pan-cytokeratin (clone PCK-26, Sigma-Aldrich Co. LLC, Saint Louis, Missouri 63103, USA, #C-1801), and mouse-anti-podoplanin (clone LF3, Novus Biologicals, Cambridge, UK, #NB110-96423).

Heat-induced antigen retrieval was performed in a citrate-buffered solution. All slides were rinsed with Tris-buffered saline (pH 7.6) plus 0.01% Tween®20 (Merck KGaA, Darmstadt, Germany, 8.22184). We incubated the slides for 20 minutes at 21°C in normal goat serum (Vector Laboratories Inc., Burlingame, CA, USA, S-1000) and then with the primary antibody for 1 hour at 21°C. As secondary antibodies, we applied a biotin-SP-conjugated AffiniPure goat-anti-mouse IgG (Jackson Immunoresearch, West Grove, PA, USA, 111-065-100) for a 30-minute incubation time at 21°C.

Immunostaining was done with a routine method using alkaline phosphatase streptavidin-biotin (Vector Laboratories Inc., Burlingame, CA, USA, #S-5100) and Fast Red as chromogen (Fast Red substrate pack, BioGenex, Freemont, CA, USA, #HK182-5 K). The slides were finally counterstained with Mayer’s hematoxylin (Linaris Biologische Produkte GmbH, Wertheim-Bettingen, Germany, EGH3411). We performed cover slipping using Aquatex® aqueous mounting medium (Merck KGaA, Darmstadt, Germany, #1.08562). Sample permeabilization, antibody concentrations, antibody reactions, and staining procedures were optimized for each antibody to get clear and specific immunohistochemical signals.

### Statistics

We compared the numbers of rats with and without mesothelioma in the different dose groups with the medium controls by the chi-squared fourfold test, chosing a level of significance of p ≤0.05.

## Abbreviations

μm: Micrometer
CNT: Carbon nanotubes
i.p: intraperitoneal
MWCNT: Multi-walled carbon nanotube
Nm: Nanometer
SEM: Scanning electron microscopy
SWCNT: Single-walled carbon nanotube
WHO: World Health Organization
